# Impact of the COVID-19 Pandemic on Emergency Department Practices for Cardiopulmonary Symptoms

**DOI:** 10.3390/jcm15020458

**Published:** 2026-01-07

**Authors:** Ki Hong Kim, Jae Yun Jung, Hayoung Kim, Joong Wan Park, Yong Hee Lee

**Affiliations:** 1 Department of Emergency Medicine, Seoul National University Hospital, Seoul 03080, Republic of Korea; emphysiciankkh@gmail.com (K.H.K.); mogu13@naver.com (H.K.); zzibii@naver.com (J.W.P.); snuemt@gmail.com (Y.H.L.); 2 Research Center for Disaster Medicine, Seoul National University Medical Research Center, Seoul 03080, Republic of Korea

**Keywords:** pandemic, emergency department, cardiopulmonary symptom, medical imaging, ultrasound

## Abstract

**Objectives:** The purpose of this study was to evaluate the trends and changes in the time to medical imaging in the emergency department (ED) for patients with cardiopulmonary symptoms during the coronavirus disease 2019 (COVID-19) pandemic. **Methods:** The retrospective observational study was conducted from the clinical database of a tertiary academic teaching hospital. Patients with cardiopulmonary symptoms (chest pain, dyspnea, palpitation and syncope) who visited an adult ED between January 2018 and December 2021 were included. The primary outcome was the time to medical imaging, including chest X-ray (CXR), chest computed tomography (CT), and focused cardiac ultrasound (FOCUS). The primary exposure was the date of the ED visit during the COVID-19 pandemic (from 1 March 2020 to 31 December 2021). **Results:** Among the 28,213 patients, 17,260 (61.2%) were in the pre-COVID-19 group, and 10,953 (38.8%) were in the COVID-19 group. The time to medical imaging was delayed in the COVID-19 group compared with the pre-COVID-19 group: the time to FOCUS was 9 min, the time to CXR was 6 min, and the time to chest CT was 115 min. **Conclusions:** We found that the time to medical imaging for patients with cardiopulmonary symptoms who visited the ED was significantly delayed during the COVID-19 pandemic.

## 1. Introduction

Cardiopulmonary symptoms are known to be among the main presenting complaints in patients visiting emergency departments (EDs) [1,2,3]. These symptoms are known to increase the risk of high severity and mortality, including economic burdens on healthcare services [4,5]. It is important for ED physicians to monitor patients with cardiopulmonary symptoms intensively because of the possibility of cardiovascular emergencies [6]. Additionally, clarifying the etiology as soon as possible is crucial since early treatment may improve the prognosis of cardiovascular emergencies [7,8,9].

Medical imaging plays a pivotal role in this diagnostic process. Chest radiography (CXR), computed tomography (CT), and focused cardiac ultrasound (FOCUS) are routinely utilized to evaluate a broad range of cardiopulmonary pathologies. The time to medical imaging in the ED is known to be important for the proper management of emergency patients, especially those with cardiovascular emergencies. Reducing the elapsed time interval between the visit to the ED and CXR or CT has been suggested to potentially improve not only ED overcrowding but also clinical outcomes [10,11]. Early FOCUS may also improve diagnostic accuracy and the rate of prescribing appropriate treatment [12]. As ED crowding and operational efficiency have increasingly been recognized as determinants of patient safety and quality of care, reducing the time to medical imaging for patients with cardiopulmonary symptoms could be essential in advancing ED practice.

The novel coronavirus disease 2019 (COVID-19) pandemic began globally in March 2020. Many EDs faced the challenge of treating patients suspected of having COVID-19 because of a lack of dedicated facilities and equipment, including negative-pressure isolation rooms [13,14]. Also, they were required to simultaneously maintain care for non–COVID-19 emergencies. In the prehospital stage, ambulance transportation for emergency calls was delayed due to the increased volume of calls [15] with infection control procedures and hospital entry restrictions in South Korea [16,17]. Patients with cardiopulmonary symptoms were reluctant to be admitted to the ED due to the risk of contracting COVID-19 [18]. Previous research has highlighted the challenges associated with medical imaging services in the ED during the COVID-19 pandemic, including delays in door-to-electrocardiogram time and reductions in imaging volume [19,20]. However, the delay of medical imaging for patients with cardiopulmonary symptoms has not yet been well evaluated.

We hypothesized that during the COVID-19 pandemic, the time to medical imaging in the ED would be prolonged, except for the time to FOCUS. The purpose of this study was to evaluate the trends and changes in the time to medical imaging in the ED for patients with cardiopulmonary symptoms during the COVID-19 pandemic.

## 2. Materials and Methods

### 2.1. Study Design, Setting, and Data Sources

This retrospective observational study was conducted on the basis of data from the clinical database of a tertiary academic teaching hospital in a metropolitan city in South Korea. The study institution is a designated regional emergency center in a metropolitan city, covering six administrative districts out of a total of 25. Approximately 70,000 emergency patients visit the hospital annually. All patients are assessed by an experienced nurse at the entrance to the ED to evaluate the severity of their condition on the basis of the Korean Triage Acuity Scale (KTAS), which consists of structured interviews and core physical examinations of vital signs. Emergency physicians routinely assess assigned patients by initially obtaining the patients’ medical histories and performing a physical examination, followed by laboratory tests and medical imaging as necessary. Routine echocardiography, which usually takes at least 2 h after the order is administered, can also be conducted. If a patient complains of cardiopulmonary symptoms, such as chest pain, dyspnea, palpitation, or syncope, FOCUS can be performed by an emergency physician. Bedside FOCUS is usually performed by either an emergency medicine residency program at the study institute [21] or the emergency board physician assigned to the patient.

The clinical database was constructed on the basis of data retrieved from the clinical data warehouse (CDW) of the study institute. The database includes patient demographic data, administrative data, clinical information before the ED visit and at the ED entrance, main diagnosis, medical imaging results with the timing of the test, FOCUS results with the timing of the test, ED medical procedures, and endpoint after ED practice.

### 2.2. Study Population

Patients with cardiopulmonary symptoms who visited an adult ED between January 2018 and December 2021 were included. Cardiopulmonary symptoms, including chest pain, dyspnea, palpitation and syncope, were defined as the main chief complaint recorded by the ED triage nurse. Pediatric patients under 18 years of age were excluded.

### 2.3. Outcome Measures

The primary outcome was the time from ED presentation to medical imaging, including CXR, chest CT, and FOCUS. The secondary outcomes were time metrics in ED practice, including the time from symptom onset to ED presentation, the time from ED arrival to triage, and the length of stay in the ED. All time points were collected from the CDW of the study institute.

### 2.4. Measurements and Variables

The primary exposure was the date of the ED visit during the COVID-19 pandemic (from 1 March 2020 to 31 December 2021). The first case of COVID-19 in Korea was reported on 20 January 2020 [22]. Considering that the effects of COVID-19 could take several weeks to appear and the declaration of the global pandemic by the World Health Organization (WHO), March was selected as the starting point of the pandemic in this study.

Demographic and clinical data, including age, sex, chief complaint (chest pain, dyspnea, palpitation, syncope, or other), method of administration (private ambulance, emergency medical service), mental status at the time of triage in the ED (alert, verbal response, pain response, and unresponsiveness), physiological status at the time of triage in the ED (blood pressure, heart rate, respiratory rate, body temperature, and pulse oximeter), and the KTAS score, were collected. In EDs in South Korea, triage is divided into five levels based on the need for medical resources: immediate resuscitation, emergency, urgent, standard, and nonurgent. The main diagnosis, excluding the lack of a definite diagnosis at discharge, was categorized as in-hospital cardiac arrest, acute coronary syndrome, congestive heart failure, cardiomyopathy, pulmonary embolism, valvular disorder, arrhythmia, infection, bleeding, cancer, or chronic disease. ED administrative data, including endpoint (admission, discharge from the ED, death in the ED, or transfer to another hospital) and intensive care unit (ICU) admission, were collected. For FOCUS examinations, the components routinely included assessing pericardial effusion, systolic dysfunction, regional wall motion abnormalities (RWMAs), and inferior vena cava (IVC) status. Timing variables (Time to imaging test and ED process) in this study were based on documented order and confirmation timestamps.

### 2.5. Statistical Analysis

Descriptive analysis was performed to evaluate the distribution of variables among the study groups. Categorical variables were compared using the chi-square test, and continuous variables were compared using the Wilcoxon rank-sum test. The primary and secondary outcomes were calculated quarterly for each year and visualized in graphs. Multivariable linear regression models were constructed for each imaging modality, with time to imaging as the dependent variable, adjusting for prespecified clinical covariates (age group, gender, triage level, chief complaint, vital sign, and ICU admission). Additionally, patients who underwent FOCUS were analyzed in terms of FOCUS findings and in-hospital information. All analyses were performed using R version 3.5 (R Foundation for Statistical Computing, Vienna, Austria).

## 3. Results

During the study period, data were retrieved and analyzed for a total of 28,213 patients. There were 17,260 (61.2%) patients in the pre-COVID-19 group and 10,953 (38.8%) patients in the COVID-19 group. Among patients presenting with cardiopulmonary symptoms, patients presenting with chest pain as the chief complaint increased in the pandemic era (49.4% vs. 44.1%) (Table 1).

The percentage of patients who underwent FOCUS increased after the onset of the pandemic (12.0% vs. 8.5%). However, the time to FOCUS decreased by approximately 9 min. Although the proportions of CXRs and chest CT scans remained similar, the time to image acquisition increased by approximately 6 min for CXRs and 115 min for chest CT scans. During the pandemic, the proportion of patients who underwent intubation and vasopressor support increased, whereas the rate of emergency coronary angiography remained nearly unchanged (Table 2).

**Figure 1:**
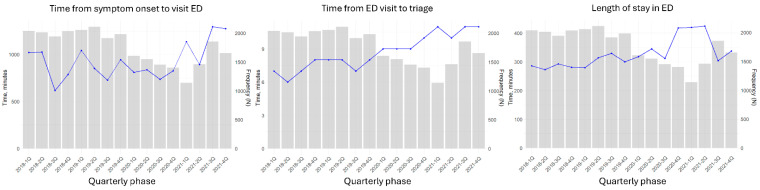
Time component from symptom onset to emergency department discharge of patient with cardiopulmonary symptom according to COVID-19 period. Abbreviations: ED, emergency department.

**Figure 2:**
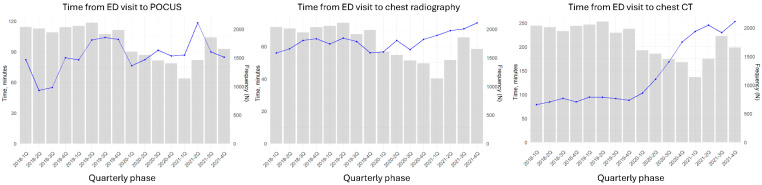
Time to assess of patient with cardiopulmonary symptoms according to COVID-19 period. Abbreviations: ED, emergency department; POCUS, point-of-care ultrasound; CT, computed tomography.

After the onset of the pandemic, the number of ED visits markedly decreased but began to increase after one year. While the length of stay in the ED initially increased and subsequently decreased, both the time from symptom onset to ED presentation and the time from ED arrival to triage continued to increase (Figure 1). Compared with chest CT, FOCUS was not significantly delayed during the initial evaluation process in the ED (Figure 2).

In multivariable linear regression analyses, the COVID-19 period was independently associated with significantly longer time to medical imaging: Beta coefficient [95% CI] 42.8 min [26.7–59.0] for POCUS, 20.8 min [17.1–24.6] for CXR, and 154 min [145–162] for chest CT, compared with the pre-COVID-19 period (all *p*-value < 0.001).

Among patients who received FOCUS, a greater proportion had the highest triage level, received endotracheal intubation, and used vasopressors, with a lower proportion having acute coronary syndrome (Table 3).

## 4. Discussion

By using data from the clinical database of a single tertiary academic hospital ED, we found that the time to chest CT for patients with cardiopulmonary symptoms in the ED was significantly shorter during the COVID-19 pandemic than the time to CXR or FOCUS. Patients with cardiopulmonary symptoms tended to visit the ED later after symptom onset, and the triage waiting time and length of stay in the ED were also prolonged. Physicians tended to apply FOCUS for patients with more severe clinical features and who were less likely to be diagnosed with acute coronary syndrome. These findings should be considered when developing diagnostic guidelines and protocols for patients with cardiopulmonary symptoms during pandemics.

There may be several reasons for the delays in medical imaging within the ED during the COVID-19 pandemic. First, the pretriage screening procedures for fever and respiratory symptoms likely contributed to prolonged waiting times between ED registration and triage [23]. In particular, patients presenting with cardiopulmonary symptoms were often assigned to isolation areas because of the difficulty in excluding the possibility of COVID-19. In such cases, depending on institutional policy, access to medical imaging could be delayed or limited. At the study institution, CXR was performed in the same manner for COVID-19 patients as for non-COVID-19 patients, with the equipment covered by plastic sheets. There were also cases where a dedicated portable CXR machine was placed inside the isolation room for patients suspected of having COVID-19. In contrast, a 30 min ventilation and disinfection period was required after the use of CT machines for patients in whom COVID-19 had not yet been excluded by polymerase chain reaction (PCR) or rapid antigen testing. These protocols likely contributed to limitations in imaging resources within the ED [24]. As illustrated in Figure 2, delays gradually increased over the study period. This may be attributable to progressive ED crowding associated with the increasing number of confirmed COVID-19 patients as pandemic evolved, resulting in overall delays in ED clinical workflows. In patients presenting with cardiopulmonary symptoms, delays in diagnostic imaging may postpone definitive diagnosis and timely initiation of appropriate therapies, and such delays could plausibly contribute to clinical deterioration and adverse outcomes, especially in time-sensitive conditions.

In contrast, FOCUS can be performed immediately with only the probe covered by a protective sheath, making it relatively unaffected by such restrictions [25]. This operational convenience may have led emergency physicians to rely more frequently on FOCUS during the pandemic (12.0% vs. 8.5%).

However, patients in the COVID-19 group who underwent FOCUS tended to be more critically ill than those in the pre-COVID-19 group who underwent FOCUS. During the COVID-19 pandemic, ED staff were often hesitant to engage closely with patients presenting with cardiopulmonary symptoms because of concerns about personal safety [26]. Consequently, clinicians may have been more inclined to rely on laboratory testing or CT imaging rather than promptly performing clinical evaluations for patients with cardiovascular emergencies. For similar reasons, FOCUS may have been less frequently performed in patients with acute coronary syndrome.

Delays in ED flow and inpatient bed shortages may have led to longer lengths of stay in the ED. Nevertheless, in hemodynamically unstable patients, delays in imaging were unacceptable, likely prompting more active use of FOCUS. These findings suggest that the utilization of FOCUS during the COVID-19 pandemic may have been influenced by selection bias.

During the COVID-19 pandemic, the proportion of patients presenting with chest pain as their chief complaint increased. This may be due to restricted ED access for patients with accompanying dyspnea and fever, whereas those with transient palpitations or syncope may have chosen to stay at home and observe their symptoms. Similarly, many patients may have delayed their ED visit until it was unavoidable, which could have contributed to an increased proportion of endotracheal intubation or vasopressor support in the ED.

On the basis of the findings of this study, FOCUS has the potential for expanded utilization in resource-constrained settings such as settings with resource constraints during a pandemic. FOCUS may also serve as a routine assessment tool during patient surges, supporting triage, diagnosis, and clinical decision-making. Potential strategies to enable such applications include wider deployment of ultrasound machines to physicians and clinicians during disaster situations, as well as implementation of modified ED practice workflows that are distinct from routine operations. Further simulation studies and protocol development research are warranted to evaluate the effectiveness of these interventions, and large-scale clinical studies should be conducted to validate their clinical utility.

### Limitations

This study has several limitations. First, the study population was selected on the basis of the chief complaint recorded by the ED triage nurse. The patients with cardiopulmonary symptoms usually had several symptoms simultaneously, and they were excluded if they had the chief complaint of altered mentality or general weakness. These limitations could be significant limitations, but we aimed to evaluate the cohort from the perspective of the ED practice flow. Additionally, the severity in the study cohort seemed to be less severe in general for this reason. Since FOCUS may preferentially performed in clinically unstable or high-acuity patients, and not all determinants of FOCUS utilization were fully captured in the dataset, residual selection bias is likely and cannot be completely excluded. Second, the time point of each medical image was collected from the CDW, which was confirmed when the assigned nurse checked the order. It may introduce potential measurement bias influenced by provider behavior and documentation practices, especially during the pandemic. Next, we could not obtain further detailed information for the study cohort after admission to the ICU or ward due to the limitations of the database. Although statistically significant delays in medical imaging were observed, particularly during the COVID-19 period, this study did not directly assess their association with patient-centered outcomes such as mortality, ICU admission, or length of stay, limiting conclusions regarding their clinical impact. Fourth, as a single-center study conducted in a large tertiary hospital within the South Korean healthcare system, our findings may not be directly generalizable to EDs with different institutional policies, triage systems, imaging workflows, or national healthcare structures. Finally, since this was a retrospective observational study, there may be unmeasurable confounders.

## 5. Conclusions

During the COVID-19 pandemic, medical imaging was delayed for patients with cardiopulmonary symptoms, with the longest delay observed for chest CT. As timely imaging is crucial for accurate diagnosis and management, especially in cardiopulmonary emergencies, advanced imaging protocols should be considered during infectious disease outbreaks to prevent delays in care.

## Figures and Tables

**Table 1:** Demographics and triage findings of patients with cardiopulmonary symptoms during the COVID-19 period.

		Study Group		
	Total	Pre-COVID-19	COVID-19	*p*-Value
Total	28,213	17,260 (61.2)	10,953 (38.8)	
Age, year, median [IQR]	65 [51, 75]	65 [52, 75]	64 [48, 75]	<0.01
Age group, years				<0.01
18~44	5375 (19.1)	2994 (17.3)	2381 (21.7)	
45~64	8630 (30.6)	5354 (31.0)	3276 (29.9)	
65~79	10,118 (35.9)	6410 (37.1)	3708 (33.9)	
80~	4090 (14.5)	2502 (14.5)	1588 (14.5)	
Male, gender	15,936 (53.0)	9792 (53.0)	6144 (53.0)	0.33
Mode of entry				<0.01
Primary visit	6189 (21.9)	4094 (23.7)	2095 (19.1)	
Transfer	2112 (7.5)	1465 (8.5)	647 (5.9)	
Ambulance				<0.01
Public EMS	6512 (21.7)	4279 (23.2)	2233 (19.3)	
Private ambulance	2206 (7.3)	1522 (8.2)	684 (5.9)	
Symptom onset to visit, minute, median [IQR]	913 [139, 4319]	864 [122, 4317]	967 [166, 4320]	<0.01
Vital sign at the ED visit				
SBP, mmHg, median [IQR]	141 [124, 165]	141 [124, 165]	142 [125, 165]	<0.01
DBP, mmHg, median [IQR]	81 [70, 92]	80 [70, 91]	82 [71, 93]	<0.01
HR, per min, median [IQR]	86 [73, 103]	86 [73, 102]	86 [73, 103]	0.49
RR, per min, median [IQR]	18 [16, 22]	18 [16, 22]	18 [16, 20]	<0.01
BT, celcius, median [IQR]	36.5 [36.3, 36.9]	36.5 [36.3, 36.7]	36.7 [36.3, 37]	<0.01
SpO_2_, %, median [IQR]	97 [95, 98]	97 [95, 98]	97 [95, 98]	<0.01
SBP below 90 mmHg	409 (1.4)	279 (1.6)	130 (1.2)	<0.01
SBP over 180 mmHg	3946 (14.0)	2355 (13.6)	1591 (14.5)	<0.01
BT over 37.5 celcius	1946 (6.9)	1070 (6.2)	876 (8.0)	<0.01
SpO_2_ below 90%	1412 (5.0)	901 (5.2)	511 (4.7)	0.04
Mental status at the ED visit				0.11
Alert	27,084 (96.0)	16,589 (96.1)	10,495 (95.8)	
Verbal response	803 (2.8)	480 (2.8)	323 (2.9)	
Pain response	237 (0.8)	147 (0.9)	90 (0.8)	
Unresponsiveness	89 (0.3)	44 (0.3)	45 (0.4)	
Chief complaint				<0.01
Chest pain	13,024 (46.2)	7615 (44.1)	5409 (49.4)	
Dyspnea	11,780 (41.8)	7410 (42.9)	4370 (39.9)	
Palpitation	1381 (4.9)	869 (5.0)	512 (4.7)	
Syncope	2028 (7.2)	1366 (7.9)	662 (6.0)	
Triage level				<0.01
Resuscitative	1259 (4.5)	715 (4.1)	544 (5.0)	
Urgent	6881 (24.4)	4525 (26.2)	2356 (21.5)	
Emergent	16,279 (57.7)	10,241 (59.3)	6038 (55.1)	
Visit to Triage, minute, median [IQR]	8 [5, 13]	7 [5, 11]	10 [6, 16]	<0.01

Abbreviations: IQR, interquartile range; EMS, emergency medical services; SBP and DBP, systolic and diastolic blood pressure; HR, heart rate; RR, respiratory rate; BT, body temperature; SpO_2_, pulse oximetry; ED, emergency department.

**Table 2:** In-hospital clinical findings of patients with cardiopulmonary symptoms during the COVID-19 period.

		Study Group		
	Total	Pre-COVID-19	COVID-19	*p*-Value
Total	28,213	17,260 (61.2)	10,953 (38.8)	
Main diagnosis				<0.01
Acute coronary syndrome	2633 (9.3)	1634 (9.5)	999 (9.1)	
Arrhythmia	1308 (4.6)	811 (4.7)	497 (4.5)	
Bleeding	305 (1.1)	199 (1.2)	106 (1.0)	
Cancer	4304 (15.3)	2740 (15.9)	1564 (14.3)	
Cardiac arrest	39 (0.1)	18 (0.1)	21 (0.2)	
Cardiomyopathy	193 (0.7)	121 (0.7)	72 (0.7)	
Heart failure aggravation	1956 (6.9)	1271 (7.4)	685 (6.3)	
Chronic disease aggravation	739 (2.6)	465 (2.7)	274 (2.5)	
Infection or sepsis	1798 (6.4)	1185 (6.9)	613 (5.6)	
Pulmonary embolism	304 (1.1)	145 (0.8)	159 (1.5)	
Unspecified shock	32 (0.1)	18 (0.1)	14 (0.1)	
Heart valvular disease	294 (1.0)	189 (1.1)	105 (1.0)	
Coronavirus infection	1375 (4.9)	56 (0.3)	1319 (12.0)	<0.01
ED evaluation				
FOCUS	2774 (9.8)	1459 (8.5)	1315 (12.0)	<0.01
Time to FOCUS, min, median [IQR]	84 [46, 154]	80 [44, 143]	89 [46, 175]	<0.01
CXR	19,170 (67.9)	11,804 (68.4)	7366 (67.3)	0.05
Time to CXR, min, median [IQR]	69 [44, 93]	61 [43, 88]	67 [45, 102]	<0.01
Chest CT	9545 (33.8)	5904 (34.2)	3641 (33.2)	0.10
Time to Chest CT, min, median [IQR]	110 [61, 227]	89 [53, 158]	204 [82, 388]	<0.01
ED resuscitation				
Endotracheal intubation	421 (1.5)	203 (1.2)	218 (2.0)	<0.01
Emergent coronary angiography	160 (0.6)	97 (0.6)	63 (0.6)	0.95
Cardiopulmonary resuscitation	25 (0.1)	8 (0.0)	17 (0.2)	<0.01
Vasopressor or inotropes	1242 (4.4)	678 (3.9)	564 (5.1)	<0.01
ED length of stay, min, median [IQR]	311 [200, 523]	295 [196, 455]	354 [208, 709]	<0.01
ED endpoint				<0.01
Discharge	17,980 (63.7)	11,084 (64.2)	6896 (63.0)	
Admission	8184 (29.0)	5001 (29.0)	3183 (29.1)	
Transfer	1921 (6.8)	1126 (6.5)	795 (7.3)	
Expire	123 (0.4)	47 (0.3)	76 (0.7)	
ICU admission	2810 (10.0)	1720 (10.0)	1090 (10.0)	0.99
In-hospital mortality	745 (2.6)	411 (2.4)	334 (3.0)	<0.01

Abbreviations: ED, emergency department; FOCUS, focused cardiac ultrasound; IQR, interquartile range; CXR, chest x-ray; CT, computed tomography; ICU, intensive care unit.

**Table 3:** Ultrasound findings and in-hospital information of patients with cardiopulmonary symptoms assessed by focused cardiac ultrasound during the COVID-19 period.

		Study Group		
	Total	Pre COVID-19	COVID-19	*p*-Value
Total	2774	1459 (52.6)	1315 (47.4)	
Time to FOCUS, min, median [IQR]	84 [46, 154]	80 [44, 143]	89 [46, 175]	<0.01
FOCUS findings				
Pericardial effusion	245 (9.2)	144 (10.3)	101 (7.9)	0.04
Systolic dysfunction				<0.01
Ejection fraction > 40%	2184 (81.8)	1098 (78.6)	1086 (85.4)	
Ejection fraction 30–40%	256 (9.6)	183 (13.1)	73 (5.7)	
Ejection fraction < 30%	229 (8.6)	116 (8.3)	113 (8.9)	
RWMA				<0.01
No definite RWMA	2289 (85.8)	1245 (89.1)	1044 (82.1)	
Regional hypokinesia/akinesia	325 (12.2)	120 (8.6)	205 (16.1)	
Global hypokinesia/akinesia	55 (2.1)	32 (2.3)	23 (1.8)	
Inferior vena cava collapse	33 (1.2)	8 (0.6)	25 (2.0)	<0.01
Inferior vena cava plethora	360 (13.5)	185 (13.2)	175 (13.8)	0.74
Triage level				<0.01
Resuscitative	210 (7.6)	81 (5.6)	129 (9.8)	
Urgent	795 (28.7)	446 (30.6)	349 (26.5)	
Emergent	1585 (57.1)	858 (58.8)	727 (55.3)	
CXR	1930 (69.6)	1045 (71.6)	885 (67.3)	0.02
Time to CXR, min, median [IQR]	67 [49, 97]	67 [51, 92]	68 [46, 105]	0.41
Chest CT	1113 (40.1)	481 (33.0)	632 (48.1)	<0.01
Time to Chest CT, min, median [IQR]	121 [63, 271]	89 [52, 152]	178 [76, 396]	<0.01
Main diagnosis				<0.01
Acute coronary syndrome	347 (12.5)	217 (14.9)	130 (9.9)	
Cardiomyopathy	34 (1.2)	12 (0.8)	22 (1.7)	
Heart failure aggravation	416 (15.0)	226 (15.5)	190 (14.4)	
Infection or sepsis	121 (4.4)	57 (3.9)	64 (4.9)	
Pulmonary embolism	49 (1.8)	17 (1.2)	32 (2.4)	
Unspecified shock	9 (0.3)	4 (0.3)	5 (0.4)	
Heart valvular disease	61 (2.2)	35 (2.4)	26 (2.0)	
ED resuscitation				
Endotracheal intubation	73 (2.6)	20 (1.4)	53 (4.0)	<0.01
Coronary angiography	21 (0.8)	14 (1.0)	7 (0.5)	0.28
Cardiopulmonary resuscitation	6 (0.2)	0 (0.0)	6 (0.5)	0.03
Vasopressor or inotropes	199 (7.2)	79 (5.4)	120 (9.1)	<0.01
ICU admission	444 (16.0)	229 (15.7)	215 (16.3)	0.68
In-hospital mortality	103 (3.7)	34 (2.3)	69 (5.2)	<0.01

Abbreviations: FOCUS, focused cardiac ultrasound; RWMA, regional wall-motion abnormalities; CXR, chest x-ray; CT, computed tomography; ED, emergency department; ICU, intensive care unit.

## Data Availability

The data presented in this study is available on request from the corresponding author.
